# The Use of Picture Cards to Elicit Postgraduate Dental Student Feedback

**DOI:** 10.3390/dj6020007

**Published:** 2018-03-30

**Authors:** Peter Fine, Albert Leung, John Francis, Chris Louca

**Affiliations:** 1 UCL Eastman Dental Institute, London WC1X 8WD, UK; albert.leung@ucl.ac.uk; 2 Centre for Higher Education Research and Practice, London KT2 7LB, UK; jfrancis86@hotmail.com; 3 University of Portsmouth Dental Academy, Portsmouth PO1 2QG, UK; chris.louca@port.ac.uk

**Keywords:** feedback, picture cards, postgraduate dental education

## Abstract

The aim of this study was to elicit information about the use of picture cards to stimulate student feedback following a postgraduate dental course. Twenty-nine general dental practitioners (GDPs) volunteered for the study. Following an explanation of how this style of feedback worked, students were asked to choose a picture card from those available and use that image to stimulate their feedback. An independent interviewer introduced the topic and gathered comments in the form of qualitative data, generated by pre-formed questions. 93% (*n* = 26) questionnaires were completed and returned. 77% (*n* = 20) GDPs reported enjoying giving their feedback by ‘picture card’ technique; 83% (*n* = 20) reported this form of feedback stimulated their thoughts. Qualitative data were analysed thematically. Some GDPs felt the picture cards helped them formulate their feedback, others felt being able to give feedback to a third party they did not know was beneficial and giving feedback as a group was helpful. This novel approach of using picture cards to stimulate feedback was well received by these GDPs. A mixed result as to the value of this style of feedback was evident. A group feedback session facilitated by a stranger was considered to be a valuable approach to take.

## 1. Introduction

The need to elicit feedback from students reporting to teachers about their learning experiences and from teachers to students about the students’ progress, understanding and future learning needs is essential [1]. In this study, we investigate a novel method for eliciting feedback provided by students to their teachers, using picture cards to facilitate that feedback. The traditional questionnaire or tick box evaluation methods, which attempt to measure participants’ reactions to learning interventions and are often designed to satisfy educational quality assurance requirements, in many cases do not adequately reflect the whole learning experience [2]. There is a perceived need for an alternative approach to producing such required feedback. The managerial measuring of learning has become a mantra or Holy Grail [3] in schools, colleges and higher education institutions. There is a belief that measuring students’ perceptions of their learning experience on a linear scale will lead automatically to improvements in teachers’ knowledge of the experience and will be helpful in future programme development [4]. Traditional student evaluations have employed a very limited range of instruments to generate qualitative and quantitative data in the form of linear scales, Likert style questions, semi-structured questions with choice of answers, multiple choice questions or preference scales [5]. Traditionally, these questionnaires have been delivered as hard copy versions, but latterly on-line versions have become more popular [6,7]. These online versions are often popular as much of the data analysis is done by the associated software of the package (e.g., Survey Monkey). These online questionnaires allow the enquirer to potentially target a wider audience, to save postal costs of a paper-based questionnaire and to instantly receive simple statistical analysis.

The various traditional feedback protocols (e.g., questionnaires, focus groups or interviews) have limitations [8], so it is incumbent upon those seeking feedback to encourage data collection from different sources and methods. There has been a tendency to treat feedback as a single notion that must be understood [9]. This present paper identifies that there is more than one idea of feedback struggling for wider acceptance. Indeed, there are several definitions of feedback, but two that are particularly applicable in postgraduate dental education and reflect the views of the student and the tutor are: (i) the means by which a student is able to gauge at each stage of the course how he or she is doing in terms of the knowledge, understanding and skills that will determine his or her result in the course [10]; and (ii) specific information about the comparison between a trainee’s observed performance and a standard, given with the intent to improve the trainee’s performance [11]. Over recent years, the language of feedback has gained greater prominence. Initially, it was used to refer to information provided by teachers to students about their work, but now feedback in the opposite direction, from students to teachers, about their learning experience has become as important. When examining recent ‘National Student Survey’ results, a consistently large minority (17%) of students have shown dissatisfaction with the amount and the quality of feedback in Higher Educational Institutions [12].

Image consumption in recent times has become so compelling that it cannot be ignored but the incidence of research in the literature to support this is somewhat lacking [13]. Human communication has been revolutionized. For this generation of students, oral and written communication are in decline. New forms of communication are now prevalent in learning environments inside and outside of the academy/institute. Communication by image has emerged as a dominant player in social, cultural and pedagogical interactions [14].

Picture card elicitation employs a range of real, figurative and abstract images [15,16]. Picture card elicitation methods were used in this manner in a discourse analysis of participants on a dental master’s programme. Picture card evaluation seeks to extend the normal range of students’ perceptions of the teaching and learning environment that is traditionally used in quality assurance surveys. 

The aim of this study was to look at a new initiative, in postgraduate dental education, for eliciting feedback from students, following a one year part time educational experience. The first year of a 4–5 year master’s programme in Restorative Dental Practice (RDP) was considered to be a suitable postgraduate student sample to trial this new approach to feedback. 

## 2. Method

**Figure 1:**
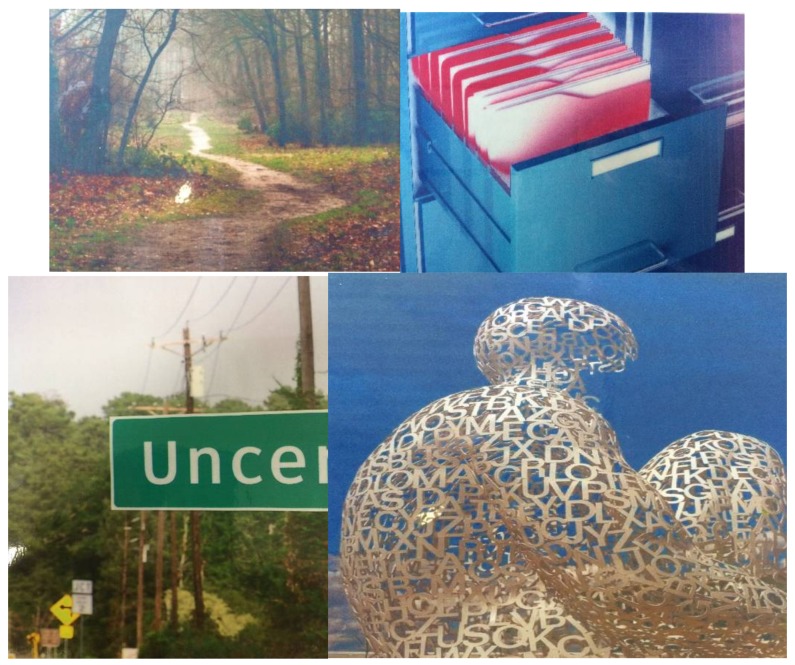
Some examples of the images shown to participants. The first image of a path through a forest can be interpreted as a clear pathway through the course; a pathway which has no ending or in which the trees are blocking the wider view. The second image of a filing cabinet can be interpreted as a neat storage place for knowledge; the open file could indicate ongoing work, or this could be one drawer amongst several that compartmentalise learning. Image three shows the uncertainty of what lies ahead; what does the sign actually say, and in what direction are we heading? Image four can be interpreted as a transparent body, where the learning taking place is transparent, students can see through the body of knowledge and see exactly what they are getting.

This study investigated postgraduate dental students’ perceptions of the use of picture cards to elicit their feedback on completion of the first year of a postgraduate dental programme. This study has been determined (by the authors) as a surface evaluation, which does not require ethics committee approval. Two feedback sessions were led by an external researcher, unknown to the students (JF), who had a background in visual art and art history and undertook the visual representation sessions. After an initial introduction, participants were asked to choose a card, which most closely represented their individual learning experience. Typically, the images included animals, landscapes, people, objects, sculpture, art, abstract and fantasy images. Participants chose images that triggered their feedback response and were asked to explain why their chosen image had generated that response. This apparently random nature of choice meant that any particular image could mean different things to different participants; there was no definitive meaning for each image, only the participant’s interpretation. For example, one participant would choose a picture card of a single pathway in a forest. The chosen image would trigger a holistic response. The response was given verbally to the whole class of participating students. Two cohorts of participants, 2015 (*n* = 15) and 2016 (*n* = 14), were recruited for this study (*n* = 29). The sessions took place during the teaching day at the end of the year, during lunchtime. This ensured that as many students as possible would be available. Teaching is carried out in small groups with a maximum of 15 individuals. However, it is undesirable to hold what amounts to a focus group with more than 12–15 participants [5]. Each participant was encouraged to choose one ‘picture card’ image from 27 available images (see Figure 1), address their peers with their reflections of the learning experience, and reflect on their choice of card.

Following this event, a short, hard copy questionnaire was designed and used to find out participants’ perceptions of this novel approach to feedback. The questionnaire enquired about (i) levels of enjoyment in giving feedback, (ii) reasons for choosing that particular card, (iii) preferred method for feedback, and (iv) preference for giving feedback as a group or individually. Quantitative and qualitative data were collected for this study.

## 3. Results

**Figure 2:**
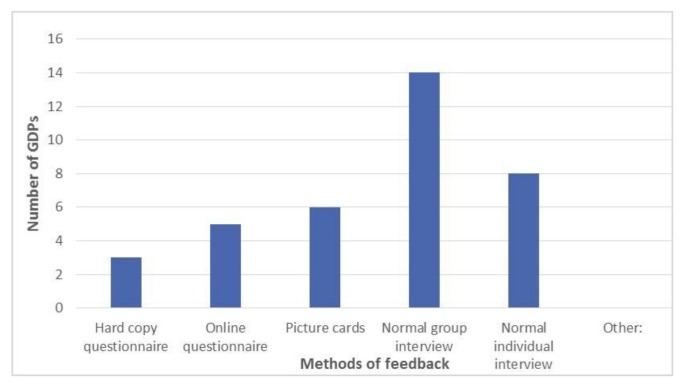
Illustrates which method of delivering feedback was reported to be most effective?

**Figure 3:**
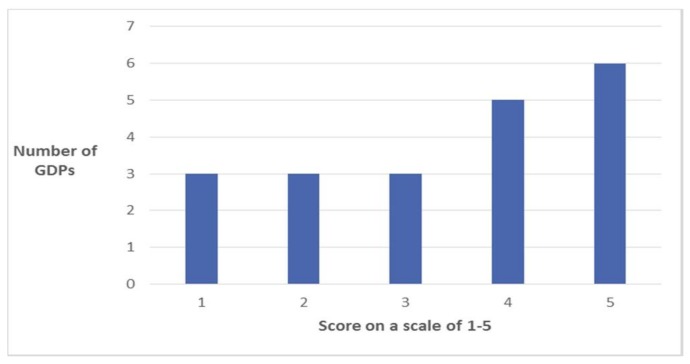
Illustrating, via a Likert scale (where 0 = not at all helpful and 5 = extremely helpful), how helpful the picture cards were in generating feedback.

Twenty-six questionnaires were returned, representing a 93% completion rate. Although not asked in the questionnaire, the postgraduate students were 59% female, age range was 25–45 years, with 63% being under 30 years of age; 78% of the students were UK trained at undergraduate level. (Additional data collected by PF). 54% (*n* = 14) of respondents reported that a group interview was the most effective method of delivering feedback (see Figure 2). The majority of respondents felt that picture cards were helpful in generating feedback (see Figure 3).

77% (*n* = 20) of GDPs reported enjoying giving their feedback by ‘picture card’ technique and 83% (*n* = 20) reported that this form of feedback stimulated their thoughts. 42% (*n* = 11) preferred to give feedback to a stranger rather than someone they knew; 76% (*n* = 19) were happy to give their feedback in a group environment. 67% (*n* = 16) reported that the picture card technique had been helpful.

Qualitative data included the following:

It was interesting; helped me get into zone
Good means of reflection and applying feedback
I felt it wasn't going to alter my ability to give feedback
I don't think it helped discussion, I would just prefer to say my opinion
It was slightly random but good selection of pictures

These comments indicated that individuals within the cohort were divided as to whether they enjoyed this new form of delivering their feedback: from the individual who found it a positive experience that helped them with feedback, to the GDP who appeared to have decided on their feedback already and was not going to be influenced by a picture card. The diversity of opinion may be due to the novelty aspect of this approach, which may change if future feedback sessions are conducted in a similar fashion.

**Figure 4:**
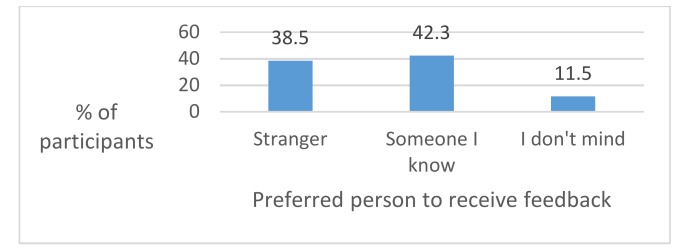
When asked who their preferred person would be to give feedback to about the course, a stranger or a known person, the majority of participants agreed that someone they know would be their preference (see Figure 4).

There was little evidence to suggest that participants in this study preferred to give their feedback to a stranger rather than an individual they know. A small percentage did not mind who they spoke to (see Figure 4).

Giving feedback to a stranger means it becomes anonymous
Using a stranger, you can be honest about some negatives of the course
Feedback to a stranger—I can be more honest
I would prefer to discuss it with someone we have met before
Feedback to someone I know, as I feel it will be taken on board more

The first three comments are examples of the qualitative data collected from those individuals who felt that they would prefer to be interviewed by a stranger. This brings into question the issue of ‘insider research’, which depending on your viewpoint can be advantageous or disadvantageous. The small majority who indicated that they either did not mind who they were interviewed by or would prefer to be interviewed by someone they know indicated that insider research can be advantageous. In this study, a lecturer unknown to the GDPs carried out the interviews, indicating the GDPs’ general preference for someone they know, as they felt more comfortable talking to them. This preference is indicated in the last two comments.

When asked about whether the GDPs preferred their feedback as a group or individually, 69.2% (*n* = 18) indicated a preference for feedback as a group.

Can feed off each other and it’s good knowing that others had same experiences
Maybe it is easier to discuss one by one
I prefer both group feedback and individual; stimulates discussion, some issues better discussed in private
Feedback as group stimulates ideas
Feedback as a group—gives you the chance to offer different opinions/agree

**Figure 5:**
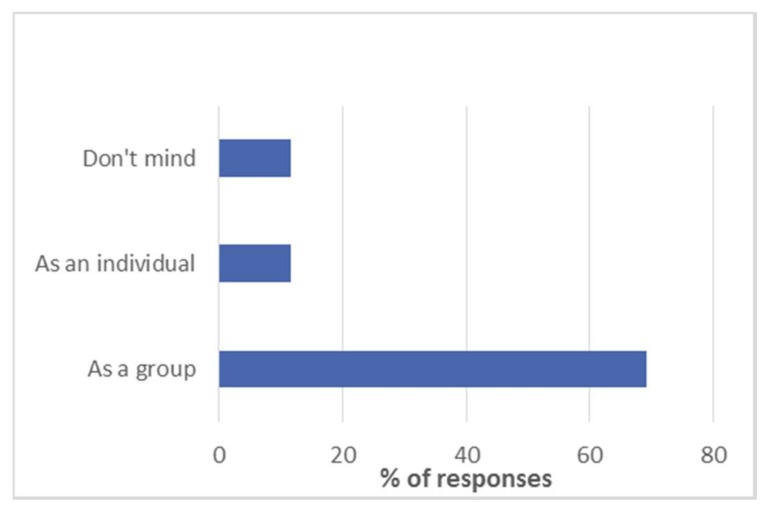
Illustrates that the participants preferred to deliver their feedback as a group rather than individually. A small number of participants did not mind whether they were part of a group or not.

This selection of comments represents the qualitative data offered by the GDPs in answer to a question about whether they preferred feedback as a group or individual. We can see that the comments support the quantitative data in Figure 5. The majority felt that there were advantages of giving feedback as a group, but we need to respect the personal nature of feedback as seen by some GDPs.

**Figure 6:**
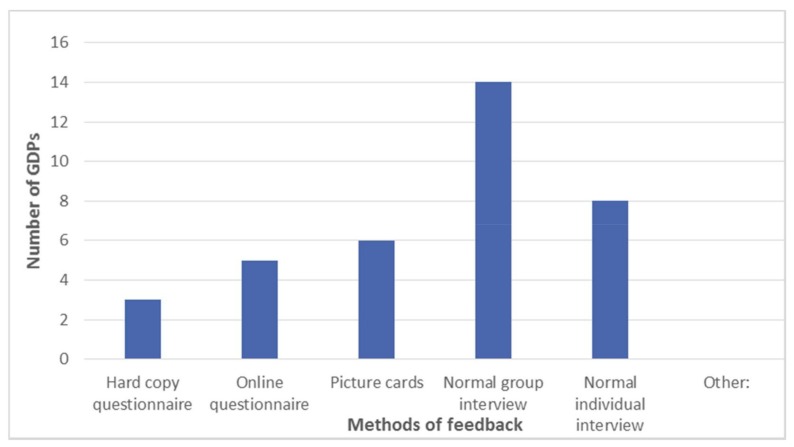
Illustrates the preferred method of delivering feedback as reported by the participants. It was clear that the majority reported being part of a group interview was preferable (see Figure 6).

When asked about which method the GDPs preferred to give their feedback, it was clear that 54% (*n* = 14) considered giving feedback via a normal group interview was their preferred method (Figure 6). Although the picture cards had stimulated thought and discussion, only 23% (*n* = 6) thought that this was their preferred method of giving feedback (Figure 6). 

Qualitative data collected included the following comments:

I think it is all about peoples’ opinions. I don't think pictures help.
Feedback as group stimulates ideas
Feedback as a group gives you the chance to offer different opinions/agree

These comments were typical of the views of the GDPs and indicated that the need for stimulation by the picture cards to give feedback was not deemed to be of any significant advantage for this cohort. In fact, it was clear that the individuals felt that the group session itself was a sufficient stimulant, as they fed off each other to give their views.

I feel lost in this forest and am finding it difficult to find my way out. The trees are stopping me from understanding what I need to know.
I felt the pathway drawn out in this picture is kind of the way that the course is structured. There is a clear pathway you can start with the certificate and take a break and carry on the Diploma and eventually the MSc and it also meant that it followed the pathway that I wanted to take in terms of my academic career and my practice career

These comments about the same picture card (See Figure 1), show the diverse opinions that are elicited from the same image. The first comment is rather negative in its inference. The student is lost in the forest and sees only the trees all around them. In the second comment, the student sees a definite pathwaywhich gives them a sense of where they are going on their postgraduate education journey. 

## 4. Discussion

Research on personally-sensitive topics such as health, gender and sexuality now use image elicitation to generate nuanced, candid and personalized responses. The results of image elicitation can be a profound aid to data collection and interpretation processes. In education, feedback and evaluation are equally sensitive areas for participants in higher education. The outsider independent nature of the feedback sessions allowed for an open and frank discussion on the quality of the learning experience of the participants. The use of the picture cards partly enabled the students to reflectively articulate their learning experience both on an individual and interactive basis. This visual method of feedback proved to be at most as effective as traditional feedback methods but may be less onerous to explore. The qualitative findings reinforced the quantitative data and provided windows into the learning, professional and personnel lives of the participants. The use of picture cards is a valuable addition to current methodologies for eliciting feedback, which requires further investigation. The results presented here indicate the effectiveness of elicitation feedback in understanding a holistic perspective of the student experience. The feedback offers insights into how group evaluations can be extended in new directions.

There were some GDPs who preferred to give their feedback as a group, citing the fact that the group environment stimulated their thoughts and reflections. Others felt more comfortable with giving feedback on a one to one basis, which allowed them to express their views in confidence. It could be argued that the latter group, in favour of giving feedback individually, was a more pure form of feedback, as they did not get prompted by their peers with the risk that they would simply repeat or agree with what had already been said. The downside of this approach is that it is possible for a much more restricted form of feedback to be given, especially if it is given to an individual the GDP is familiar with. There could be an element of wanting to please the known tutor. 

The findings of this study indicated that the majority (58%) of the cohort were happy to give their feedback to someone they knew; however, this means that a significant minority were more comfortable giving feedback to a stranger. Previous studies have indicated that interviews are more productive with a known interviewer [17], but in this study several GDPs felt that they preferred being interviewed by a stranger, so that perceived potential repercussions of offering feedback to a known person could not occur. However, this cohort felt that the advantage of knowing their interviewer indicated that the points made were more likely to be taken on-board and that the individual knew what they were talking about.

The elicited picture card representations supported participants’ engagement with their current or past pedagogic experiences and generated new understanding of institutional praxis. Picture card elicitation demonstrated the embodiment of the learner experience both inside and outside the class room. The employment of images releases learners from the power of the written word which often generates and maintains distress. Participants used picture card images to generate rich metaphors and construct narratives of their learning and development. These findings have implications for enhancing interpretive dental education research by incorporating picture card elicitation methods.

The importance of fostering reflection in healthcare education is well established [13,18,19]. The use of photographs has been described as ‘mirrors which we project upon and have reflected back to us various aspects of our awareness’ [20]. Various characteristics of photographs such as abstract/concrete, static/dynamic, active/passive ‘supply a context from which meaning can evolve’ [20] and these unanticipated meanings can arise as can lasting emotional effects [21,22]. 

Future studies or extensions of this study will need to look at a more robust sample size. The sample size of 29 GDPs recruited for this study was small, and so larger cohorts will need to be included to offer more representative results. The limitations of the study included the sample size as well as the funding required to employ more ‘strangers’ to carry out the feedback group sessions, and the need to know more about the psychological aspect of picture card feedback technique. The learning environment is a complex arena that can include psychological, social, cultural, personal and pedagogic contexts [23].

## 5. Conclusions 

The novel approach of using picture cards to stimulate feedback following a year-long restorative programme was well received by the participating postgraduate dentists. The overwhelming impression was that although the cards were thought to be stimulating by some, others felt that the cards were of no real value and did not enhance their feedback. It was interesting to hear that the participants felt it was important to conduct a group feedback session, which was facilitated by a stranger to the GDPs, rather than one of their tutors. Previous feedback has been undertaken using a questionnaire collected by administration staff, but this cohort seemed to value the anonymity of their feedback, perhaps feeling they could be completely honest and were uninhibited in their responses.
